# Hearing threshold and physical performance in older people: a cross-sectional study from the HUNT4 cohort

**DOI:** 10.1007/s41999-022-00713-6

**Published:** 2022-11-18

**Authors:** Sylwia Kolasa, Bård Bogen, Roy Miodini Nilsen, Stein Helge Glad Nordahl, Frederik Kragerud Goplen, Bo Engdahl, Dara Meldrum, Jan Erik Berge, Kjersti Thulin Wilhelmsen, Pernille Thingstad, Lisa Aarhus, Liv Heide Magnussen

**Affiliations:** 1 Department of Health and Function, Western Norway University of Applied Science, Post Box 7030, 5020 Bergen, Norway; 2 Department of Otorhinolaryngology & Head and Neck Surgery, Haukeland University Hospital, Bergen, Norway; 3 Department of Clinical Medicine, University of Bergen, Bergen, Norway; 4 Department of Physical Health and Ageing, Norwegian Institute of Public Health, Oslo, Norway; 5 Department of Occupational Medicine and Epidemiology, National Institute of Occupational Health, Oslo, Norway; 6 Academic Unit of Neurology, Trinity College Dublin, Dublin, Ireland; 7 Department for Rehabilitation Services, Haraldsplass Deaconess Hospital, Bergen, Norway; 8 Department of Neuromedicine and Movement Science, Norwegian University of Science and Technology, Trondheim, Norway

**Keywords:** Older adults, Postural control, Hearing threshold, HUNT, Physical performance

## Abstract

**Purpose:**

To evaluate the association between increased hearing loss and reduced physical performance in older people.

**Methods:**

Cross-sectional population-based study using data from the fourth wave of the Trøndelag Health Survey (HUNT4) in Norway. Data were obtained from the subproject HUNT4 Hearing which collected audiometric data of people > 70 years (*N* = 13,197). Analyses were performed on all participants who had completed audiometry and measured balance using the Short Physical Performance Battery (SPPB), which was scored from 0 (worst score) to 12. The hearing threshold was expressed as a pure tone average (PTA). Associations between the hearing threshold for the best and worst ear and physical performance were analyzed by linear regression models adjusted for age, sex, education, diabetes, and cardiovascular disease. Hearing threshold was indicated with steps of 10 dB.

**Results:**

Of 13,197 eligible participants, 4101 who completed audiometry and SPPB (52.3% women. mean age 76.3 years) were included. The analyses revealed an association between reduced SPPB and increased hearing threshold in the best ear (*b* = − 0.296; 95% CI − 0.343 to − 0. 249; *P* < 0.001) and the worst ear (*b* = − 0.229; 95% CI − 0.270 to − 0.189; *P* < 0.001).

**Conclusions:**

In this population study, we found that the increased hearing threshold was associated to reduced physical performance as measured by SPPB. The association seemed to be strongest for the best ear. The association between hearing threshold and physical performance illustrates the importance of assessing physical performance in people with hearing loss to prevent the risk of falls and disability. The underlying causes of the associations between hearing loss and poorer physical performance are not fully understood and should be further investigated.

**Level of evidence:**

Level 3.

## Key summary points

**Aim:**

To evaluate the association between hearing threshold and reduced physical performance in older people.

**Finding:**

Association between a higher hearing threshold and poorer physical performance was observed.

**Message:**

It is important to assess balance and falls risk in older people with hearing loss.

## Introduction

Approximately 1.57 billion people live with hearing loss worldwide, and this is projected to increase to 2.45 billion by 2025. In 2019, hearing loss was the third leading cause of years lived with disability for all ages, and the leading cause among those who were 75 or older [1], and it is associated with reduced communication skills, psychosocial challenges, social inactivity, and high healthcare costs [2]. In a study from Norway, the prevalence of moderate hearing loss (dB 35–49) was 18.7 percent in individuals who were older than 64 years old [3].

Age-related hearing loss is associated with many factors such as long-term exposure to noise, genetic predisposition, chronic diseases (such as cardiovascular disease, diabetes mellitus, ear disease), ototoxic medications, poorer socioeconomic status and unhealthy lifestyle (e.g. smoking) [2, 4]. Very often, hearing loss in older people are not detected, as health professionals frequently ignore the hearing problem at the expense of other health problems that are considered more important and prioritized by older people [5, 6]. Hearing loss is associated with cognitive impairment [7], cardiovascular death in men [8], increased risk of depression [9], and social isolation [10]. Some studies also suggest that hearing loss is related to motor skills in older adults, such as reduced mobility and physical performance [11–13]. Although they emphasized potential biases, Jiam et al. found a two-fold increased risk of falling in older people with hearing impairment [14]. Lin and Ferrucci found that for every 10 dB decrease in the best ear, there were 40% higher odds of a self-reported fall in a cohort of older people compared to people with good hearing [15], and in a cohort of female twins, Viljanen and co-authors found that the quartile with the poorest hearing in the best ear had an incidence rate ratio of falls of 3.4 compared to the quartile with the best hearing [16]. Why there should be an association between reduced hearing and poorer physical performance is not entirely clear, but in a review, Carpenter and Campos refer to three hypotheses from the literature; (i) that problems in detecting the origin of sound make spatial orientation and interaction with the environment more difficult, (ii) that hearing loss puts more demands on attentional resources, leaving less for maintaining balance, and (iii) that there is concomitant pathology in the auditory and postural systems [17].

There is evidence to suggest that a higher hearing threshold is associated with poorer physical performance in older people [11], but further research is needed. Large population-based studies with audiometric measures of hearing and physical performance outcomes are warranted. Therefore, the aim of this study was to examine the association between hearing loss measured by audiometry and reduced physical performance measured by the Short Physical Performance Battery (SPPB) as registered in the fourth wave of the North-Trøndelag Health Survey (HUNT4) in Norway [3].

## Methods

The present study is a cross-sectional population-based study using data from HUNT4. While the proportion of people with higher educational levels and number of immigrants in this part of Trøndelag is slightly lower, the general health, unemployment rate, disability insurance, and mortality rate are close to the national average [3]. 13,197 people were invited to participate in the two subprojects of HUNT4: HUNT70+ and HUNT4 Hearing. Inclusion criteria for the present study were the availability of audiometric data and complete score of the physical performance test SPPB. The study was approved by the Regional Committee for Medical and Health Research Ethics of South-East Norway (23,178 HUNT Hearing). Participation in the study was based on an informed written consent.

### Study population

Inclusion criteria for the present study were the availability of audiometric and physical performance data based on the SPPB. The current study included 4110 people with such data participating in both HUNT70+ and HUNT4 Hearing. The data collection for HUNT4 70+ and HUNT4 Hearing took place from September 2017 until March 2019 [3]. Participants completed a self-assessment questionnaire at home and standardized interviews and clinical questionnaires were conducted at field stations, at participants' homes or at the institution where they lived. Clinical evaluation was performed by trained research assistants using standard protocols.

### Hearing threshold

Hearing was examined without hearing aids by air-conduction pure-tone audiometry at eight frequencies from 0.25 to 8 kHz. For this study, the pure tone-average (PTA) was calculated separately on each ear as the average hearing thresholds from the frequencies of 0.5, 1, 2 and 3 kHz, measured in dB hearing level (HL) as recommended by the Hearing Committee of the American Academy of Otolaryngology—Head and Neck Surgery [18]. The assessment was done by teams consisting of an audiologist and two trained assistants. The pure-tone audiometry was conducted in sound attenuation booths. Ambient sound levels at the various sites were below the ISO-criteria for test tones in the 0.25–8 kHz range. The audiometers used were type Interacoustics AD629 with TDH-39P supra-aural audiometric headphones with PN51 cushions. For a more detailed description of the procedure, please see Engdahl et al. [3].

### Physical performance

Physical performance was measured using the SPPB. The SPPB was developed to measure physical capability and to assess the lower extremity physical performance status of an older individual [19]. SPPB has been shown to predict several negative outcomes in older adults, such as all-cause mortality [20], falls [21], and disability [22]. The SPPB consists of three tasks: standing balance, gait speed, and a chair rise task. To assess standing balance, the participant is asked to maintain balance in three different positions; in the first, a patient stands with feet together side-by-side, in the second, then it is semi tandem stand with the selected foot a step forward so that the heel side of the foremost foot touches the big toe of the other, and in the third, it is full tandem stand i.e. the foot is set behind so that the heel of one foot is in front of the other foot. The test is performed in the described order, and the subsequent position is attempted only if the previous one does not cause a problem and the participant is able to hold the position for 10 s. To measure walking speed, the participant is asked to walk 4 m at a normal pace. Timing of the task starts after the command “ready–start” is given and ends after crossing a line marking 4 m. The test is repeated twice, and the better time is recorded. To test the strength of the lower limbs, the participant is asked to get up from a straight-back standard chair without the use of the upper limbs. If pre-testing of the chair rise- test is successfully completed, the participant is asked to repeat this activity five times as quickly as possible, and their time in seconds is recorded.

The participant can be awarded 0–4 points for each task, and so the total score can vary from 0 to 12. For interpretation, 0–6 is considered a low score, 7–9 a middle score, and 10–12 a high score. For a detailed description of how points are awarded, please see Guralnik et al. [23].

### Other variables

To assess the independent role of hearing on physical performance, we accounted for other variables that may have an impact on both hearing and physical performance. To select variables for adjustment, we used direct acyclic graphs (DAG) and the online software DAGitty (http://www.dagitty.net/). The DAGitty was designed to create, edit and analyze causal diagrams for minimizing bias in empirical studies in epidemiology and other disciplines [24]. Among the variables that were available to us from the HUNT4-dataset, we assumed that age, sex, education, diabetes and cardiovascular disease could have an impact on both hearing threshold and physical performance, and as such confound the association of hearing with physical performance. These variables were thus chosen as confounding factors in the multiple regression analysis.

### Analysis

All analysis was performed using Stata SE (16th edition; StataCorp, College Station, Texas). To examine the relationship between hearing threshold (independent variable) and SPPB (dependent variable), we used linear regression models. Information on sex, age, education, diabetes, cardiovascular disease, PTA variable, total SPPB score, static balance, gait speed and chair rise were quantified using descriptive statistics. The complete SPPB score, static balance, gait speed, and chair rise were outcomes under study. The PTA hearing thresholds in the better and the worse ear were the main exposures. Analyses were performed crudelu and after adjustment for the following potential confounding factors: sex, age, education, diabetes and cardiovascular diseases as independent categorical variables in the regression models.

## Results

Among 4110 participants with a total SBBP score who were included in the analyses, 9 did not have complete hearing data. To analyze the association of hearing threshold with SPPB total score, and the separate tests standing balance, gait speed and chair stand, we included 4101 participants with complete information about their age, 4067 with complete information on education, and 3846 with complete information on cardiovascular diseases and 4002 on diabetes. We had no information about how many of those included lived in their own homes and were tested at the test stations, and how many were nursing home residents. Demographic data on the study participants, and comorbidities, as well as summary statistics on hearing thresholds and SPPB scores are shown in Tables 1 and 2 Hearing threshold seems to increase with increasing age, and the gap between hearing threshold in the best and worst ear was consistent across the age groups with 6.4 in the age group 70–74 years and 6, 8 in the age groups 75–79 years and > 80 years.

**Table 1:** Distribution of PTA hearing threshold by demographics and comorbidities in 4101 participants

	No. (%)	PTA hearing threshold in dB * mean (SD) (*n* = 4101)	
		Best ear	Worst ear
Age^a^			
70–75	2017 (49.2)	20.2 (11.8)	26.6 (14.8)
76–80	1215 (29.6)	25.4 (13.1)	32.2 (15.0)
> 80	869 (21.2)	33.9 (14.1)	40.7 (15.8)
Sex			
Man	1960 (47.7)	26.1 (14.5)	33.4 (16.6)
Woman	2150 (52.3)	22.7 (12.7)	28.5 (14.9)
Education^b^			
Primary school	979 (24.1)	25.8 (13.7)	32.3 (16.0)
High school	1896 (46.6)	24.7 (13.6)	31.2 (15.9)
University	1192 (29.3)	22.6 (13.4)	29.0 (15.8)
Diabetes^c^			
No	3546 (86.6)	24.1 (13.7)	30.6 (1.60)
Yes	456 (11.4)	26.1 (13.3)	32.5 (15.5)
Cardiovascular disease^d^			
No	2976 (77.4)	24.0 (13.4)	30.4 (15.8)
Yes	870 (22.6)	25.8 (14.7)	32.6 (16.6)

*The average hearing thresholds (PTA) from the frequencies of 0.5, 1, 2 and 3 kHz

^a^Missing data *n* = 9

^b^Missing data *n* = 43

^c^Missing data *n* = 108

^d^Missing data *n* = 264

**Table 2:** Distribution of physical performance by demographics and comorbidities in 4110 participants

	No. (%)	Short physical performance battery (SPPB) (*n* = 4110)			
		Total score, (0–12) Mean (SD)	Standing balance, (0–4) Mean (SD)	Gait speed, (0–4) Mean (SD)	Chair rise, (0–4) Mean (SD)
Age^a^					
70–75	2017 (49.2)	11.0 (1.6)	3.8 (0.6)	3.8 (0.5)	3.4 (1.0)
76–80	1215 (29.6)	10.4 (2.0)	3.6 (0.8)	3.7 (0.7)	3.1 (1.1)
> 80	869 (21.2)	9.0 (2.8)	3.2 (1.1)	3.3 (0.9)	2.5 (1.4)
Sex					
Man	1960 (47.7)	10.7 (2.0)	3.7 (0.7)	3.7 (0.6)	3.3 (1.1)
Woman	2150 (52.3)	10.2 (2.2)	3.6 (0.9)	3.6 (0.8)	3.0 (1.2)
Education^b^					
Primary school	979 (24.1)	9.8 (2.5)	3.4 (1.0)	3.5 (0.8)	2.8 (1.3)
High school	1896 (46.6)	10.5 (2.1)	3.6 (0.8)	3.7 (0.7)	3.2 (1.1)
University	1192 (29.3)	10.9 (1.7)	3.8 (0.6)	3.8 (0.5)	3.4 (1.1)
Diabetes^c^					
No	3546 (86.6)	10.6 (2.0)	3.6 (0.8)	3.7 (0.7)	3.2 (1.1)
Yes	456 (11.4)	9.7 (2.7)	3.4 (1.0)	3.5 (0.9)	2.8 (1.3)
Cardiovascular disease^d^					
No	2976 (77.4)	10.6 (2.0)	3.7 (0.8)	3.7 (0.6)	3.2 (1.1)
Yes	870 (22.6)	10.1 (2.5)	3.5 (0.9)	3.6 (0.8)	3.0 (1.3)

^a^Missing data *n* = 9

^b^Missing data *n* = 43

^c^Missing data *n* = 108

^d^Missing data *n* = 264

Table 3 presents the estimated association of PTA hearing threshold in the worst and the best ear with SPPB total score. The coefficients were highest for the ear with the best hearing. Crude linear regression analysis showed that a 10 dB increase in hearing threshold in the best hearing ear was associated with 0.296 point [95% CI (− 0.343 to − 0.249)] reduction in the total SPPB score. This association remained after adjusting for sex, age and education as well as after additional adjustment for diabetes mellitus and cardiovascular disease (− 0.138; 95% CI − 0.187 to − 0.089).

**Table 3:** Association between PTA hearing threshold and SPPB total score, the HUNT4 (2017–2019) surveys, Norway (*N* = 4101)

	Crude		Model 1*		Model 2*	
	Coefficient (95% CI)	*P* value	Coefficient (95% CI)	*P* value	Coefficient (95% CI)	*p* value
Best ear	− 0.296 (− 0.343 to − 0.249)	< 0.001	− 0.140 (− 0.189 to − 0.092)	< 0.001	− 0.138 (− 0.187 to − 0.089)	< 0.001
Worst ear	− 0.229 (− 0.270 to − 0.189)	< 0.001	− 0.114 (− 0.155 to − 0.073)	< 0.001	− 0.110 (− 0.152 to − 0.069)	< 0.001

*Model 1 = Adjusted for age, sex, and education. *Model 2 = Adjusted for age, sex, and education, diabetes, and cardiovascular disease. The coefficient signifies a change in SPPB score per 10 dB change in hearing threshold by the linear regression model. Missing data: age *n* = 9, education *n* = 43, diabetes *n* = 108, cardiovascular disease *n* = 264

*SPPB* short physical performance battery

Table 3 shows the estimated association of PTA hearing threshold in the best and the worst ear with separate tests and standing balance, gait speed and chair stand. The associations seemed to be stronger for the ear with the best hearing. Crude linear regression analysis suggested that a 10 dB increase in hearing threshold in the better ear was associated with a deterioration in standing balance, in gait speed, and in chair stand. This association remained also after adjusting for sex, age and education with standing balance, with gait speed and chair stand, as well as after additional adjustment for diabetes mellitus and cardiovascular disease with standing balance [estimates with 95% CI − 0.032 (− 0.057 to − 0.012)], with gait speed [estimates with 95% CI − 0.026 (− 0.042 to − 0.010)] and chair stand [estimates with 95% − 0.075 (− 0.103 to − 0.048)] (Table 4).

**Table 4:** Association between PTA hearing threshold and standing balance, gait speed and chair rise (*N* = 4101)

	Crude		Model 1*		Model 2*	
	Coefficient (95% CI)	*P* value	Coefficient (95% CI)	*P* value	Coefficient (95% CI)	*P* value
Standing balance						
Best Ear	− 0.091 (− 0.108 to − 0.072)	< 0.001	− 0.037 (− 0.057 to − 0.018)	< 0.001	− 0.032 (− 0.057 to − 0.012)	0.002
Worst Ear	− 0.073 (− 0.088 to − 0.057)	< 0.001	− 0.034 (− 0.051 to − 0.018)	< 0.001	− 0.031 (− 0.048 to − 0.014)	< 0.001
Gait speed						
Best Ear	− 0.067 (− 0.082 to − 0.052)	< 0.001	− 0.026 (− 0.042 to − 0.009)	0.002	− 0.026 (− 0.042 to − 0.010)	0.002
Worst Ear	− 0.054 (− 0.068 to − 0.041)	< 0.001	− 0.024 (− 0.038 to − 0.011)	< 0.001	− 0.024 (− 0.038 to − 0.010)	0.001
Chair rise						
Best Ear	− 0.138 (− 0.164 to − 0.112)	< 0.001	− 0.075 (− 0.102 to − 0.047)	< 0.001	− 0.075 (− 0.103 to − 0.048)	< 0.001
Worst Ear	− 0.102 (− 0.125 to − 0.080)	< 0.001	− 0.054 (− 0.077 to − 0.031)	< 0.001	− 0.053 (− 0.077 to − 0.030)	< 0.001

*Model 1 = Adjusted for age, sex, and education. *Model 2 = Adjusted for age, sex, and education, diabetes, and cardiovascular disease. The coefficient signifies that one-unit change in standing balance per 10 dB change in hearing threshold by linear regression model. Missing data: age *n* = 9, education *n* = 43, diabetes *n* = 108, cardiovascular disease *n* = 264

## Discussion

In this cross-sectional population-based study, we examined the association of hearing threshold with physical performance in 4101 older people in the HUNT4 study, making this one of the largest studies on the topic to date. We found that a higher hearing threshold measured by audiometric tests was associated with poorer physical performance as measured by SPPB.

Our results suggested that the association between hearing threshold and physical performance was stronger for the best ear, compared with the worst ear. This is in line with the findings of Berge et al., who found a stronger association between body sway and best ear hearing in adults who attended a specialist clinic for vestibular complaints [13]. In other studies, such as those by Lin and Ferrucci [15] and Viljanen and co-authors [16], only data from the ear with the best hearing was included. The World Health Organization defines hearing loss as a hearing threshold in the best ear [25], as this best represents the person’s remaining hearing abilities. It also suggests that the best hearing ear and the function of the “healthy” side best predict the physical function. This indicates that reduced hearing threshold may be a marker of reduced physical performance in general. On the other hand, worst ear hearing includes one-sided hearing loss that is more likely related to specific insults of one ear (such as shooting noise) and thus less affected by systemic changes affecting both hearing (in both ears) including the vestibular system.

In Tables 1 and 2, we can see that the average hearing threshold in the best ear for the youngest group (70–75 years) was 20.2 dB, which is at the limit of mild hearing impairment, while the oldest group had a hearing threshold of 33.3 dB, which is nearer to a ‘disabling hearing loss’ of 35 dB or higher, according to the World Health Organization [26]. Reports from the US suggest that 68% of 70–79 years old, and 89% of those over 80 years have a bilateral or unilateral hearing threshold of 25 dB or higher [27]. It is difficult to compare the results directly, but the findings indicate that hearing impairment is highly prevalent in older populations. Interestingly, hearing has improved in Norway over the last twenty years, with higher education, less occupational noise exposure, ear infections and less smoking being probable explanations [28].

Overall, the 70–75 years group had an average total score of 11 on the SPPB, and the group that was 80 years or older had an average of 9.1 (Table 1). This is comparable to another Norwegian population-based study (the Tromsø Study) [29] and to a study of Singaporeans [30], and higher than in Colombians [31]. The data from the Tromsø Study may be most relevant for comparison with our study: In the Tromsø Study, the participants scored fairly similarly on the different tests, while the participants in our study scored lower on the chair-rising test; the male 80+ group in the Tromsø Study scored 3.04 and the women scored 2.73. The score for the men and women combined in our study was 2.6 points. The chair-rising test is the most strength-demanding of the SPPB-tests, and women seemed to have longer chair-rising time than men [32]. It is also interesting that the chair-rise time is the test with the highest coefficient in the regression analysis. This could indicate that hearing and physical performance is more strongly related to loss of muscle strength than to reduced inner ear function. In a systematic review, it was concluded that fatigue of the lower extremity and trunk muscles impairs balance and physical performance and thus lead to an increased risk of falls [33]. However, even when sex differences are considered, the participants in our study appear to score systematically lower than in the Tromsø Study for SPPB. It should be noted that in the HUNT4-study, effort was made to include also nursing home residents, while the Tromsø Study included only those who could attend the testing stations. Therefore, there may be more participants with lower physical capacity in the HUNT4 Study.

The cross-sectional observational design of the study does not allow for inferences about causality, and we do not know if hearing impairment causes loss of balance and mobility problems, or if these issues appear due to a third common causative factor. Some factors were accounted for, such as cardiovascular disease, which can affect both hearing and physical performance [34]. In our study, hearing threshold predicted balance even when controlling for cardiovascular disease and diabetes, which could lend some support to the hypothesis that high hearing thresholds causes reduced balance, and poor hearing and reduced balance are not necessarily both just natural and coinciding occurrences in the aging process or due to associated pathology. However, the presence of cardiovascular disease and diabetes are based on self-report in the HUNT study, which may have some degree of inaccuracy. Further studies with more comprehensive assessments of potential confounders are necessary to shed light on this association.

The link between dementia and hearing loss in older adults has been the focus of increasingly more research and cognitive function may therefore represent a confounder in our study. In memory clinic patients, individuals with severe hearing loss measured by audiometry had significantly more dementia than others [35]. In a report on dementia prevention, intervention and care, midlife hearing loss is the potentially modifiable risk factor that attributes to most late-life dementia [36]. We do not have information about the cognitive status of our participants, but another study has been published about the cognitive status of the HUNT 70+ -cohort [37]. Based on the entire sample (*n* = 9930), the researchers found that approximately 35 percent had mild cognitive impairment and 15 percent had dementia. We cannot say that these proportions apply to our sample, but we assume that a fair number of our participants also had dementia or mild cognitive impairment. Lack of knowledge about cognitive status limits the interpretation of our results, as associations between cognition, and disability, frailty and physical performance have been found previously [38, 39]. Cognitive status may therefore be an underlying factor that explains the association between hearing loss and physical performance. Further studies should investigate this.

Strengths of our study include the large population-based sample, objective hearing assessments and a balance test that is widely used in clinical practice. The large number of participants included in this study provides the good statistical power to detect associations. However, 13,197 people were invited to participate in both the 70+ -cohort and hearing study of HUNT4. Of these, only 4101 had audiometry and underwent the SPPB test. As such, the generalizability of our study results can be questioned. Further, we do not have information about how many of the participants were ones that lived in their own homes, and how many that were in residential care. A selection towards healthier subjects in terms of both balance and hearing loss may have underestimated the associations. Since this was a population study, we have limited data on the diagnosis of both hearing loss and reduced physical performance in individual cases. Conductive hearing loss could not be analyzed, since bone conduction thresholds were not available to us. However, in the elderly, sensorineural hearing loss is predominant, and at least unilateral conductive hearing loss can be ruled out as a major factor since physical performance was most strongly related to hearing threshold on the best hearing ear. This applies to both hearing and physical performance. Therefore, we were provided solely with data on air conduction and not on bone conduction. Tests of vestibular function could have given relevant information on some of the causes of reduced physical performance. However, previous studies (Berge et al.) indicated that poor balance even in dizzy patients may be more related to age and general physical weakness than to peripheral vestibular function as measured by the caloric test. Another limitation is that we have no information about the use of hearing aids during balance testing. Rumalla and co-authors found that physical performance was better when habitual hearing aid users wore them compared to when they did not [40, 41]. It is imaginable that hearing aid use could have had an impact on physical performance also in this study.

In conclusion, an increased hearing threshold is associated with poor physical performance in a population-based study of older people. For this reason, it is relevant to assess falls risk in older patients with increased hearing threshold due to hearing loss. Likewise, older people with reduced physical performance should be screened for symptoms of increased hearing threshold. It is not clear whether treatment of hearing loss leads to improved postural stability or physical performance. Further research is needed to investigate possible preventive measures that may improve both hearing threshold and physical performance in the aging population, and to investigate the possible causality between increased hearing threshold and physical performance. This could be researched through cohort studies or experimental studies.
